# The Time, Electric Field, and Temperature Dependence of Charging and Discharging Currents in Polypropylene Films

**DOI:** 10.3390/polym15143123

**Published:** 2023-07-22

**Authors:** Shuting Zhang, Fuqiang Tian, Jieyi Liang, Jinmei Cao, Zhaoliang Xing

**Affiliations:** 1 School of Electrical Engineering, Beijing Jiaotong University, Beijing 100044, China; 19117036@bjtu.edu.cn (S.Z.); 21121433@bjtu.edu.cn (J.L.); 21117019@bjtu.edu.cn (J.C.); 2 State Key Laboratory of Advanced Power Transmission Technology, Beijing 102209, China; 18846136536@163.com

**Keywords:** charging and discharging current, charge transport mechanism, trap level distribution, carrier mobility, charge accumulation, electric field distortion, polypropylene film

## Abstract

The insulating properties of polypropylene (PP) film play a very important role in the operating status of direct current (DC) support capacitors. Charging and discharging currents in PP film under high DC electric fields and temperatures correspond to charge transportation and accumulation, which significantly influence the electrical insulating properties of PP. In this paper, we have comprehensively studied the dependence of charging/discharging currents in PP film on time, electric field (150–670 kV/mm), and temperature (40–120 °C). The results showed that the charging current increased by almost an order of magnitude from 150 kV/mm to 670 kV/mm and exhibits a steep increase with temperature above 80 °C. The discharging currents are about 10 times less than the corresponding charging currents. Carrier mobility varies little with the electric field and becomes slightly larger with an increase in temperature. The quantity of the accumulated charges was calculated by the integral of the charging and discharging current differentials and showed a significant increase with the electric field and temperature. The corresponding electric field distortion becomes larger above 80 °C compared to 20–60 °C. Both electric field and temperature have an important effect on PP film and capacitors based on charge transport and accumulation and their electric field distortion. This study is innovative in that it combines the operating status of DC support capacitors with traditional methods to research synthetically charged transport mechanisms of PP film. The findings are meaningful for understanding the insulation failure mechanisms of PP film and capacitors under complex stresses.

## 1. Introduction

The large-capacity modular multi-lever converter (MMC) is the key equipment to realize alternating current (AC) and direct current (DC) conversion in the high-voltage direct current (HVDC) transmission system [1,2,3]. As one of the core components in the converter submodules (SMs), the performance of the DC support capacitors directly affects the stability of the transmission system. In the actual operation of MMC, all SMs need to constantly switch except for the “off” state, meaning that the capacitors in the SMs are in a constant process of charging and discharging [4,5,6,7]. DC support capacitors are subjected to high temperatures in addition to complex electrical stresses during operation [8,9]. A large number of heat-generating devices, such as insulated gate bipolar transistors (IGBTs) and DC support capacitors, lead to high temperatures in the converter valve hall. The high temperature will cause the DC support capacitor dielectric loss to increase and heat generation to increase, resulting in a further rise in temperature and increasing the probability of insulation failure of the capacitors. PP film is the main dielectric material in DC support capacitors, and it sustains high operating electric field strength and temperature. Therefore, the study of the PP film insulation failure law at high temperatures and high electric fields is of great significance for improving the insulation performance of DC support capacitors.

The interaction of high electric fields and heat is the main reason for dielectric failure. The structure of PP film undergoes significant phase changes above 80 °C. Therefore, temperature has a great influence on the characteristics of film breakdown and conduction currents [10]. When the ambient temperature is high and heat dissipation conditions are poor, the internal temperature of capacitors is high, and PP film is susceptible to breakdown, especially in the presence of overvoltage [11,12]. The charge transport in PP film at high temperatures and high electric fields is important to reveal their insulation failure mechanism. However, the physical process and the microscopic mechanism of charge transport are not clear.

In order to study the charge transport and insulation failure mechanisms of polypropylene films, and taking into account the operating conditions of capacitors constantly charging and discharging at high temperatures, we conducted charging and discharging experiments at different temperatures to simulate the electrical and thermal stresses on PP film during the normal operation of capacitors. The charging/discharging currents are important approaches for the space charge transport mechanism under high electric fields. Several authors have previously analyzed the charging and discharging currents for polypropylene films. M. Nagao et al. studied the charging/discharging currents and electric field strengths of PP films below 100 kV/mm at 100 °C [13]. The results showed that homo-space charge depressed the charging currents and caused anomalous discharging currents. Relative to the cathode metal, the anode metal largely determines the electric strengths. M. Moudoud et al. studied the charging/discharging currents of PP under 0.3 kV/mm after thermal aging at 80 °C for 3–36 days. The results showed that the charging currents increase, anomalous discharging currents often occur, and the time of the reversal current decreases with thermal aging [14]. However, the thicknesses of the PP films they studied were 18 µm, 30 µm, and 3 mm, respectively, and the films were subjected to low electric fields. The findings are not suitable for high-voltage DC capacitors. Janet Ho et al. investigated the charging current of PP at 40–100 °C. They deduced that charge transport occurs by a hopping process, with the hopping distance varying from 1.4 nm to 3.2 nm, and is field-independent [15]. Xing et al. systematically studied the charging/discharging currents and charge transport microphysical processes of polypropylene films under 150–670 kV/mm at 20 °C. They found that there was a large amount of space charge accumulation when the electric field exceeded a threshold [16]. Janet Ho et al. removed the memory effect before each voltage application. Xing et al. did not consider the effect of temperature on PP films.

The novelty of this study can be summarized as follows: (1) the comprehensive dependence of charging/discharging currents of PP film on time, electric field, and temperature, especially about space charge accumulation and electric field distortion with time under different electric fields and temperatures; (2) the charge transport mechanism and insulation failure mechanism of PP film based on actual operating conditions of DC support capacitors. The increasing electric fields accelerate the failure of PP film step by step.

In this study, we investigated the charging/discharging currents in polypropylene film under 150–670 kV/mm at 40–120 °C for 7200 s, which basically covers the various conditions in which the PP film suffered during practical operation of the capacitors. Furthermore, trap-level distribution, carrier mobility, charge accumulation, and their evolution are derived from the charging/discharging currents. Finally, we calculated the electric field distortion caused by charge transport based on the Poisson equation. The findings of this study are valuable for the development of DC support capacitors based on insulation failure mechanisms.

## 2. Materials and Methods

**Figure 1:**
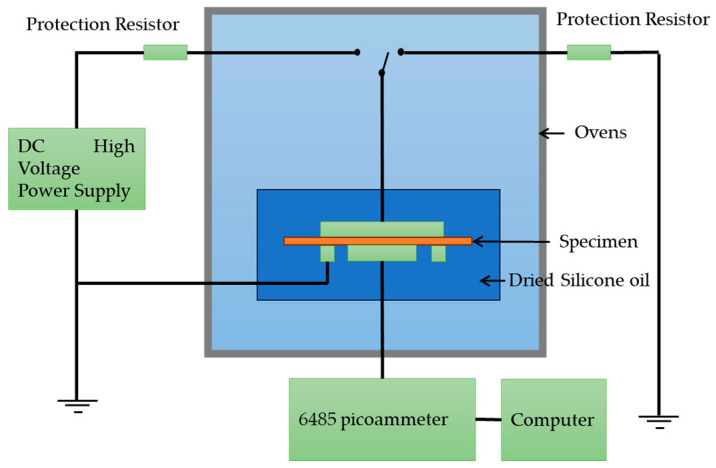
Charging/discharging currents testing system.

The specimens were biaxially oriented polypropylene (BOPP) film with a nominal thickness of 9 µm for the capacitor, produced by Anhui Tongfeng Electronics Co., Ltd. (Anhui, China) PP films, which had aluminum electrodes on both sides of the film with a diameter of 30 mm, were steamed on both sides to produce the specimens. The measurements were separately taken in the oven at 40 °C, 60 °C, 80 °C, 100 °C, and 120 °C. The initial applied electrical field on the specimen was 200 kV/mm for 7200 s, then the specimen discharged for 7200 s at a constant temperature. The next applied electrical field was 230 kV/mm; then it increased by 40 kV/mm each time until the specimen was broken by cyclic charging and discharging. The experiment was repeated at another constant temperature. Charging/ discharging currents were recorded for 2 h using a 6485 picoammeter at varying temperatures and electric fields. Only the oven temperature was controlled at the desired constant value and maintained before and during the measurement. During the isothermal experiments, specimens were kept at the desired temperature for at least 10 min and then the initial charging voltage was applied for 7200 s while the I_c_ was continuously recorded. The testing system is shown in Figure 1.

## 3. Experimental Results

**Figure 2:**
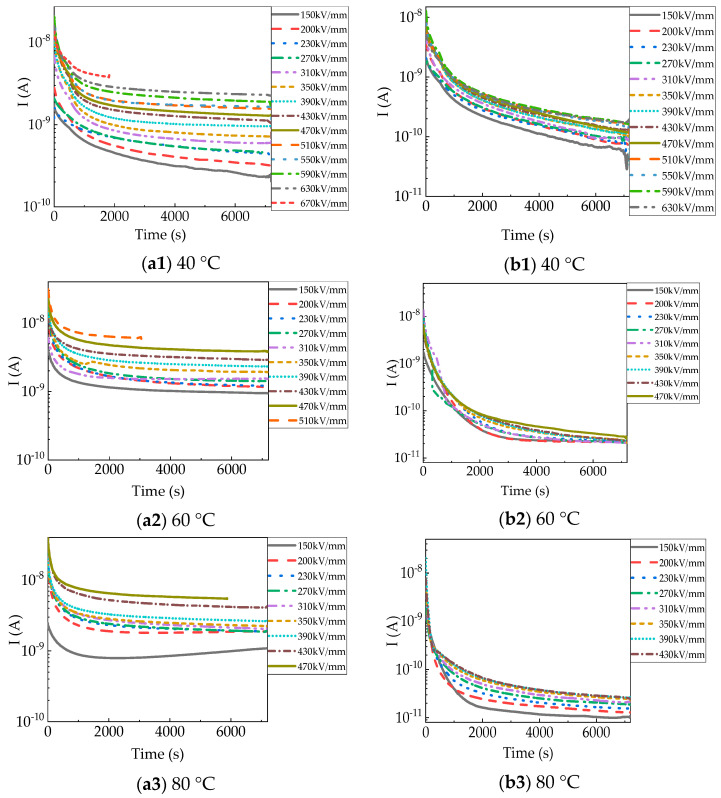

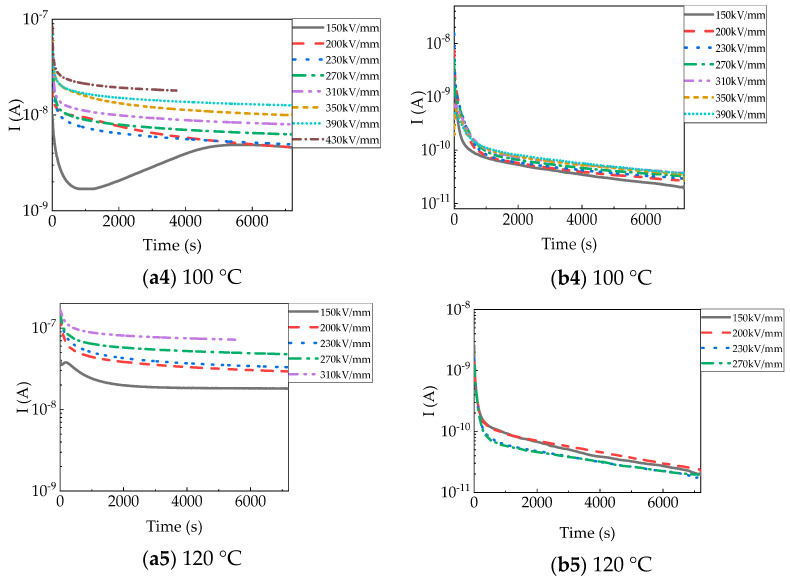
Charging and discharging currents of PP film until breakdown at different temperatures: (**a**) charging currents; (**b**) discharging currents.

Figure 2 shows the charging currents (I_c_) and discharging currents (I_d_) under varied electric fields and temperatures (40–120 °C). I_c_ and I_d_ are dependent of the time, to some extent.

**Figure 3:**
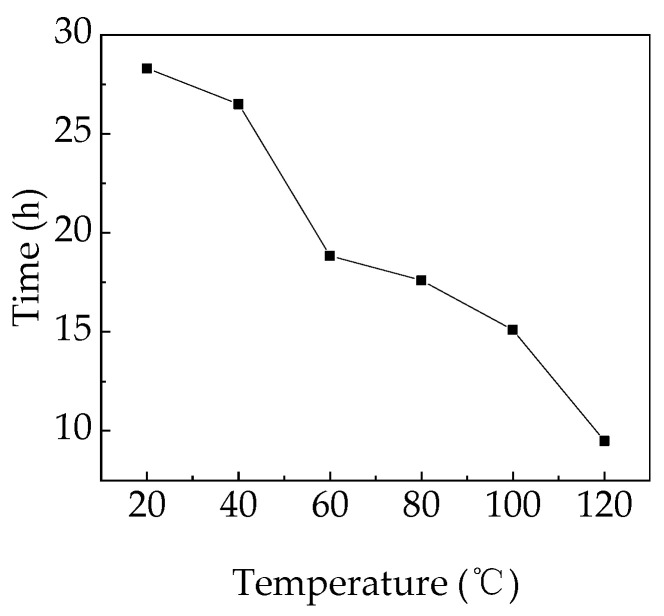
Aging life of PP films versus temperature under consecutive charging and discharging.

At 40 °C, the initial charging current (I_c_) in Figure 2(a1) was approximately 1.59 × 10^−9^ A under 150 kV/mm. I_c_ continued dropping with time to 2.28 × 10^−10^ A, and eventually did not reach a steady state. I_c_ rose with increasing electric fields and reached a steady-state value under 430 kV/mm. The initial I_c_ increased slightly to 1.59 × 10^−8^ A, and the steady-state I_c_ increased to 2.31 × 10^−9^ A under 630 kV/mm. Breakdown of the PP film occurred when the applied electric field was raised to 670 kV/mm and lasted for 1825 s. The aging life of PP films at 40–120 °C is shown in Figure 3 [16]. The I_d_ in Figure 2(b1) had the same upward trend as the electric field. The initial and final I_d_ were approximately 1.77 × 10^−9^ A and 6.23 × 10^−11^ A, respectively, under 150 kV/mm. However, the values of I_d_ in the several fields before breakdown (510–630 kV/mm) were similar, with both steady-state values being about 1.7 × 10^−10^ A. I_d_ was obviously much less than I_c_.

At 60 °C, the trend of the charging current (I_c_) in Figure 2(a2) with time and electric field was the same as at 40 °C, only the values were larger. But I_c_ reached a steady-state value under 230 kV/mm. The time for I_c_ to reach a steady state was much shorter. Steady-state I_c_ apparently increased from 9.47 × 10^−10^ A to 3.86 × 10^−9^ A as the electric field increased from 150 kV/mm to 470 kV/mm, respectively. Breakdown of the film occurred after the electric field was 510 kV/mm for 3029 s. The I_c_ before breakdown was approximately 6.01 × 10^−9^ A. After removing the voltage, the I_d_ in Figure 2(b2) dropped faster than at 40 °C and had a lower value, with a final value of approximately 2.23 × 10^−11^ A, except under 470 kV/mm.

At 80 °C, the charging current (I_c_) in Figure 2(a3) first went down and then slowly went up with time under 150 kV/mm and 200 kV/mm, which means space charge injection. When the applied voltage varied from 150 kV/mm up to 430 kV/mm, the final I_c_ increased from 1.07 × 10^−9^ A to 4.10 × 10^−9^ A. The film breakdown occurred at 470 kV/mm at 5878 s, and the corresponding current was 5.45 × 10^−9^ A. The I_d_ in Figure 2(b3) dropped sharply to 2.62 × 10^−11^ A after removing 150 kV/mm. The I_d_ dropped sharply to 3.75 × 10^−11^ A after removing 430 kV/mm, which also follows the pattern of increasing with the electric field.

At 100 °C, the charging current (I_c_) in Figure 2(a4) first went down and then went up, obviously increasing with time at 150 kV/mm, which means more space charge injection. The cause may be that the electrode injection current was smaller than the bulk conduction current or that the bulk conductivity was extremely high, which resulted in an increase in the interface electric field and lowered the charge injection potential [17,18]. The steady-state I_c_ under 150 kV/mm was a little greater than the final I_c_ at 200 kV/mm. The final I_c_ increased from 4.57 × 10^−9^ A to 1.27 × 10^−8^ A with the applied electric field increasing from 200 kV/mm to 390 kV/mm, respectively. Breakdown of the film occurred after the field was 430 kV/mm for 4000 s. The final I_d_ in Figure 2(b4) increased from 3.75 × 10^−11^ A to 1.93 × 10^−11^ A with the electric field variation. I_d_ dropped faster than at 80 °C.

At 120 °C, the final I_c_ in Figure 2(a5) was about 1.82 × 10^−8^ A at 150 kV/mm. It increased to 4.78 × 10^−8^ A at 270 kV/mm. Before breakdown, the I_c_ was 7.32 × 10^−8^ A at 310 kV/mm. The final I_d_ in Figure 2(b5) was approximately 2.09 × 10^−11^ A in the short circuit discharging process at 150 kV/mm, I_d_ was approximately 1.97 × 10^−11^ A at 270 kV/mm. The I_d_ showed no obvious trend, with the final value varying in the range of 1.97 × 10^−11^–2.49 × 10^−11^ A. At 100 °C and 120 °C, the I_c_ before breakdown was even one order of magnitude higher than the transient I_c_ under the initial electric field.

The charging current reached stable values more quickly when temperatures and electric fields were higher. At the same time, I_d_ decayed to smaller values faster. This is because at higher temperatures, the density of free carriers is higher, and carriers in shallow traps are more likely to detrap. Due to steady-state conduction current, generated traps, and so on, I_c_ and I_d_ have no mirror symmetry, and I_c_ is much larger (almost one order of magnitude) than I_d_ at high temperatures and high electric fields [19,20].

## 4. Discussion

### 4.1. Time Dependence of Charge Injection

**Figure 4:**
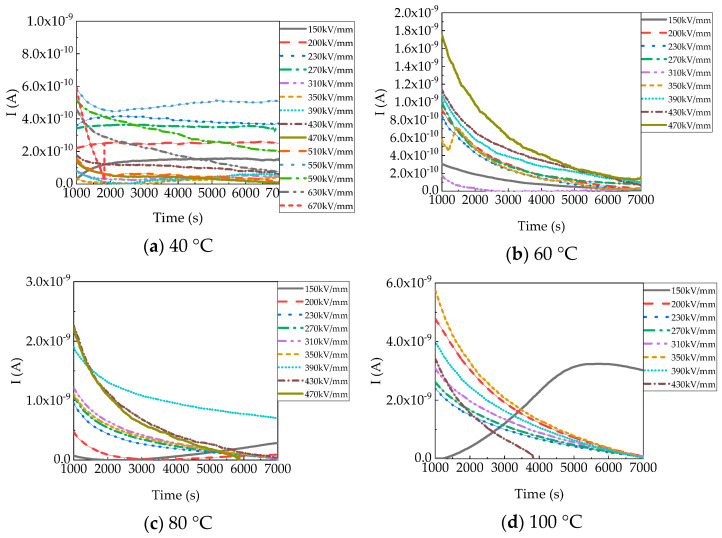

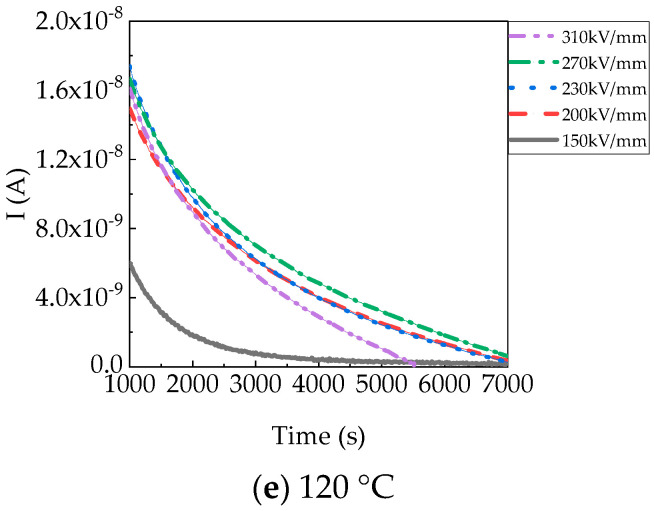
Conduction currents of PP films at different temperatures.

Figure 4 shows the currents about charge injection in PP films at different temperatures. Charging currents subtract discharging currents and conduction currents from the steady value of I_c_–I_d_. In general, the currents had a rising trend with an increasing electric field. Compared with currents under 150 kV/mm, currents under high electric fields were higher, which can be fundamentally caused by the decrease of the carrier injection barrier and the increase of carrier mobility. Carrier transport and changes in carrier mobility will be discussed later. The injected charges trapped around the electrode interface did not form a sufficient trapped charge layer, which contributed to space charge injection and higher bulk conductivity under 150 kV/mm at 80 °C and 100 °C. Therefore, the currents rose with time [17]. Moreover, temperature has a profound effect on currents. It is easier to reach steady-state currents in PP films at 40 °C. When the temperature increased, the initial current became larger and dropped faster, and the carrier injection rate became faster. This is because the high temperature promoted the emergence of high-energy electrons and increased their free path [21]. This also means the electric properties of PP film are unstable at higher temperatures.

### 4.2. Electric Field and Temperature Dependence of Charging Currents

**Figure 5:**
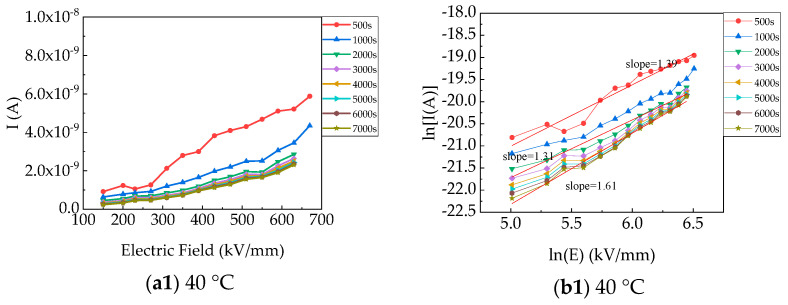

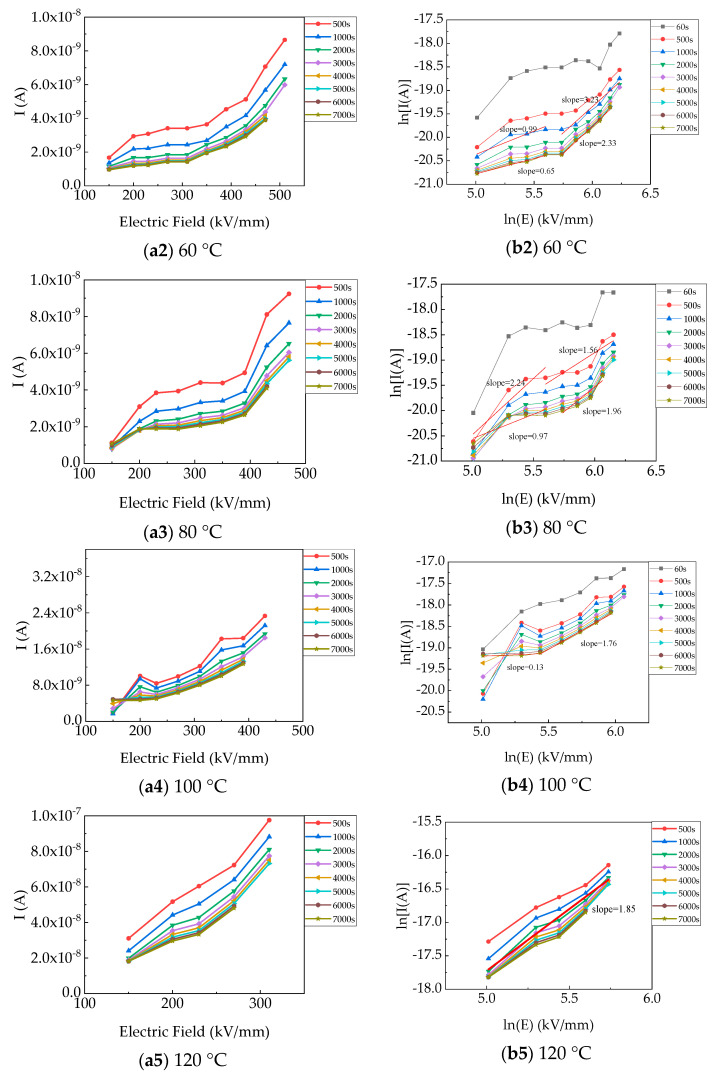
Charging currents of PP films versus electric field at different temperatures: (**a**): *I-E*; (**b**) *lnI-lnE*.

Figure 5 shows the charging current (I_c_) versus the electric field at 40–120 °C. It is clear from Figure 5a that the I_c_ increased with an increase in the electric field at the same temperature. The upward trend under different electric fields was basically the same at 2000–7000 s. The increase in electric fields caused the I_c_ to increase to very high transient values and subside to much smaller steady–state values a few minutes later. The increased electric fields forced more charges into the conduction band, which contributed to the increased transient currents. Then, most of the free charge settled into traps, so that the current decayed rapidly [22]. The charge transformation process was accelerated with an increase in the electric field.

**Figure 6:**
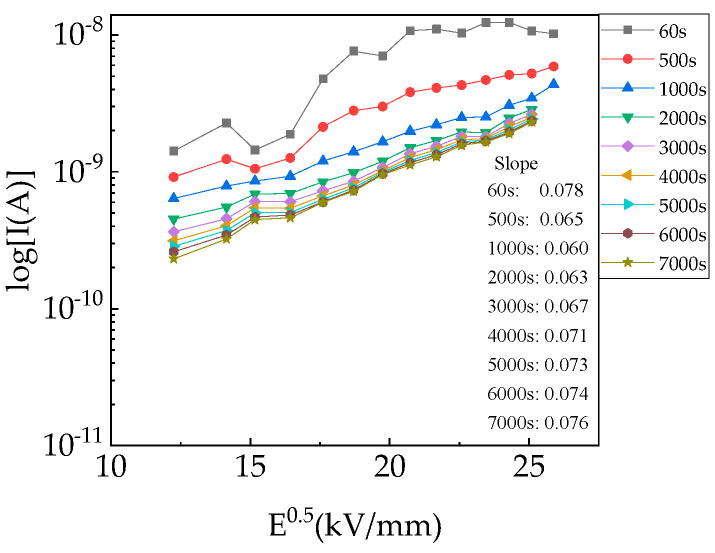
Charging currents of PP film versus *E*^0.5^ at 40 °C.

Figure 5b shows the charging current (I_c_) versus the electric field at 40–120 °C in the form of *lnI–lnE*. Under almost all the applied electric fields, the *lnI–lnE* did not display any Ohmic behavior (the slope was +1). The slope deviated from Ohmic behavior and increased linearly or non-linearly. According to several classical conduction mechanisms [23,24], data fitting was used to explain the dependence of I_c_ on the electric field. Based on the plot of *log I–E^0.5^* (a straight line) within 0.060–0.078 at 40 °C in Figure 6, the dielectric constants were too high for PP films (2.25) [15,25]. Therefore, Poole–Frenkel and Schottky mechanisms were not dominant conduction mechanisms. The Fowler–Nordheim mechanism was not the dominant conduction mechanism due to the slope of *log (J/V*^2^) − 1/*V*, which was a positive number (*J*: current density; *V:* voltage). Space-charge-limited conduction (SCLC) was also eliminated in that the square relationship of *lnJ–lnV* was not true. And based on Figure 4, *lnI* and *lnE* exhibited a piecewise linear relationship, which might represent the change in the hopping distance with increasing electric field. Therefore, the hopping conduction mechanism is reasonable to further analyze the conduction properties [26]. There is an increasing trend in hopping distances with temperatures [27].

**Figure 7:**
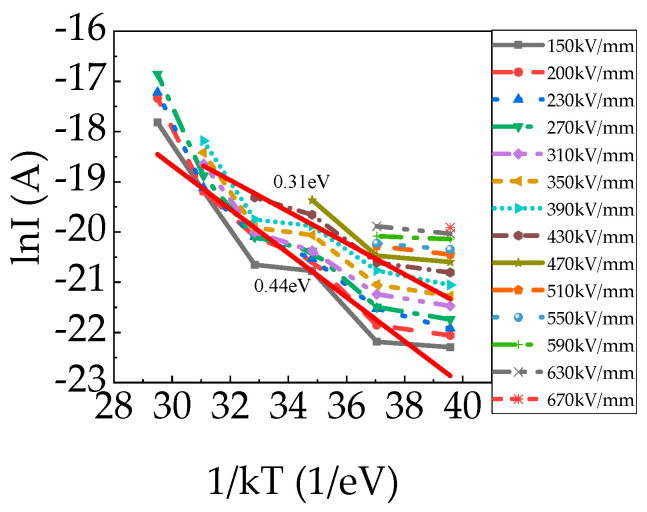
Charging currents (7000 s) of PP film versus temperatures under different electric fields.

Figure 7 shows charging currents versus temperatures under different electric fields at 7000 s. In general, I_c_ increased exponentially with temperature at 20–120 °C. The activation energy was approximately 0.31–0.44 eV regardless of the applied electric fields, indicating that conduction mechanisms were the same within 150–670 kV/mm. Samples measured at different temperatures were not the same sample, but the data can be used as a reference. It is obvious that the activation energy at 20–120 °C was divided into four stages. A dramatic increase was observed above 40 °C and 80 °C, which means a large amount of space charge was trapped in the PP film at 40 °C and 80 °C. It is consistent with the thermally stimulated current (TSC) of BOPP films, which exhibited three peaks at approximately 45, 78, and 120 °C [28]. That is to say, carriers trap in deeper traps in the crystalline and/or at the crystalline/amorphous interfaces and/or in impurity/defect traps in the amorphous region at higher temperatures.

**Figure 8:**
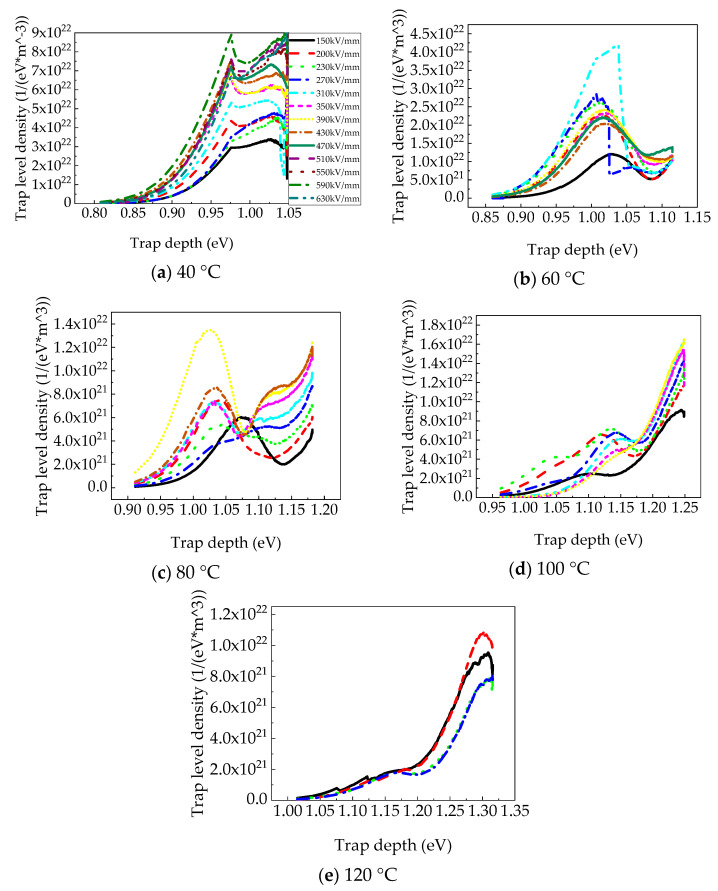
Trap level distribution of PP films at different temperatures.

Figure 8 shows the trap-level distribution of PP films at different temperatures based on calculation methods proposed by Tian [29]. Obviously, the trap density increased significantly with an elevated electric field at a constant temperature, and the trap depth hardly changed. The lower the temperature, the more strictly this rule was followed. This may be due to the fact that, at the same temperature, the trapped charge, whose depth was smaller than the trap depth corresponding to the peak of the trap density, accounted for a large proportion of the total trapped charge, the trap cross section was large, the charge trapping probability was large, and the increased electric field would further promote the charge trapping. The activation energy at 40 °C exhibited two peaks (approximately 0.98 eV and 1.03 ± 0.01 eV), as shown in Figure 8a. The activation energy at 60 °C was 1.02 ± 0.02 eV, as shown in Figure 8b. The trap distribution at 80 °C also exhibited two peaks (approximately 1.02 eV and 1.13 eV) when the electric field was higher than 230 kV/mm, as shown in Figure 8c. The activation energy at 80 °C changed irregularly. The activation energy at 100 °C, as shown in Figure 8d, was 1.13–1.14 eV, except for 1.10 eV at 150 kV/mm. The activation energies were separately 1.16 eV and 1.31 eV at 120 °C, as shown in Figure 8e. As the temperature increased, the trap depth charges increased, but the trap density tended to decrease because charges trapped in the shallow traps were difficult to trap. On the one hand, a trapped charge has a shorter lifetime due to the higher probability of detrapping caused by excitation, and, on the other hand, the charge is trapped more easily in deep traps during charge transportation with temperature [30].

### 4.3. Electric Field and Temperature Dependence of Discharging Current

**Figure 9:**
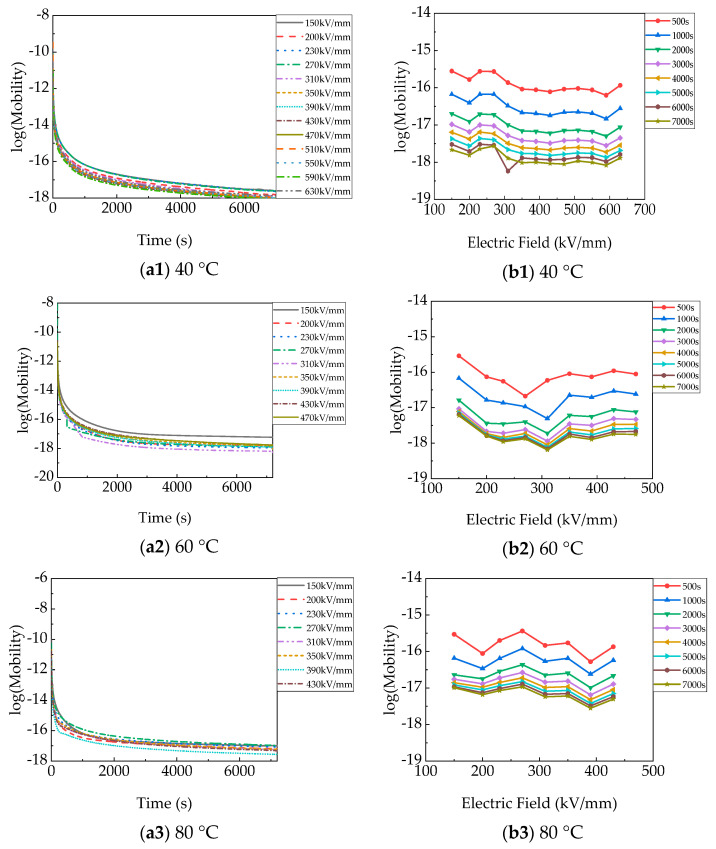

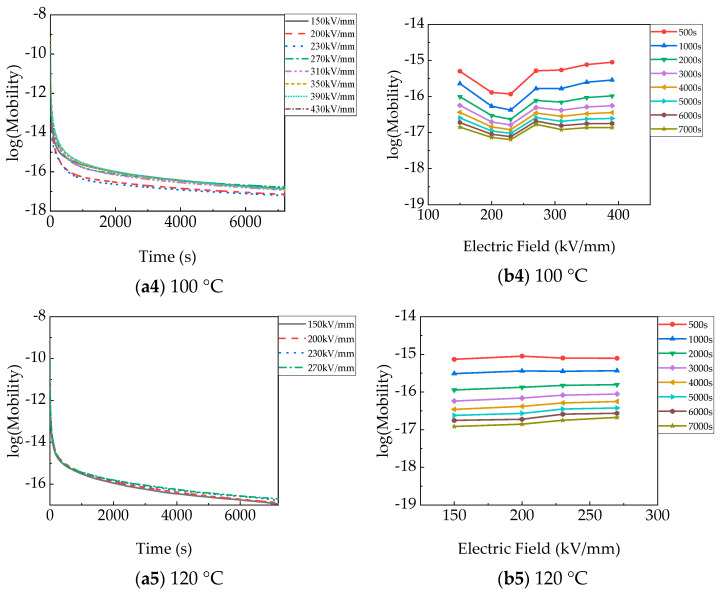
Charging currents of PP films versus time (**a**) and electric field (**b**) at different temperatures.

Figure 9 shows the carrier mobility of polypropylene films at 40–120 °C and 150–630 kV/mm, which is derived from the isothermal short-circuit discharge current [29,31]. It is known that the mobility of PP films decreases with time, which includes two stages at all temperatures. In stage 1, the mobility index decreases with time, and the initial mobility is very high. Mobility drops slowly with time in stage 2. The two stages are related to the fast decay of free charge and the detrapping of space charge in shallow and deep traps, respectively, in a short circuit. It is consistent with the trend and mechanism of I_d_. At 40 °C, mobility took the lead with a slight decrease and then slowly increased with the electric field, as shown in Figure 9(a1,b1). At 7000 s, it was almost 10^−18^ m^2^ /V·s. At 6000 s, the sharp decline at 310 kV/mm was an accidental condition in the experiment. At 60 °C, 80 °C, and 100 °C, the mobility decreased and then increased with the elevated electric field. But mobility increased with increasing temperatures. It was almost 10^−17^ m^2^ /V·s at 100 °C and 7000 s. The mobility increased slightly with the elevated electric field at 120 °C, as shown in Figure 9(a5,b5), and it was approximately 10^−17^ m^2^/V·s at 7000 s, slightly greater than at the other temperature.

Generally speaking, the higher the electric field and temperature, the more likely the carriers in deep traps are to detrap; the carriers in shallow traps are easy to detrap; the free carriers migrate faster, so the carrier mobility is greater. Mobility is related to the progressive emptying of free charge and trapping charge. There are fewer traps for carriers to be trapped in with increasing temperatures and electric fields, which results in elevated carrier mobility.

### 4.4. Time, Electric Field, and Temperature Dependence of Charge Accumulation

**Figure 10:**
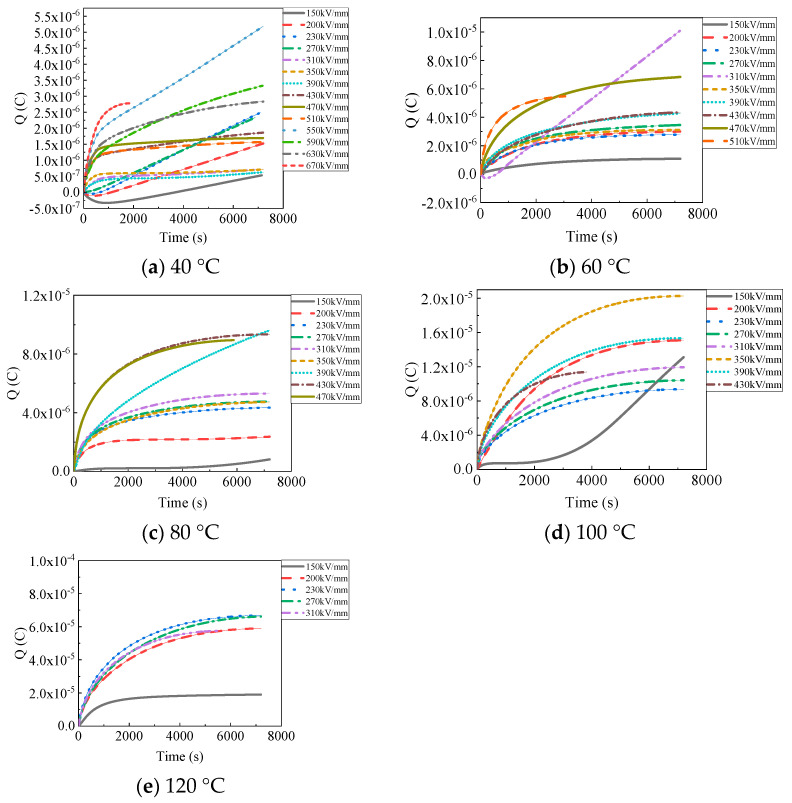
Charge accumulation of PP films versus electric field at different temperatures.

Figure 10 shows space charge accumulation (Q) in PP films during continuous pressurized charging and discharging at different temperatures. Space charge accumulation (Q) was obtained by integrating the conduction currents in Figure 3 over time. At the same temperature, charge accumulation generally increased with an increasing electric field, but did not strictly follow the rule [32,33]. When the applied electric field was 150 kV/mm for 7200 s at 40 °C, as shown in Figure 10a, the Q was 5.3 × 10^−7^ C. The Q was, respectively, 2.5 × 10^−6^ C and 7.1 × 10^−7^ C at 230 kV/mm and 310 kV/mm for 7200 s. When the applied electric field increased to 630 kV/mm for 7200 s at 40 °C, the Q was 2.8 × 10^−6^ C, much greater than the Q at 150 kV/mm. Larger values of Q were obtained at 60 °C and 80 °C. The Q was, respectively, 1.1 × 10^−6^ C and 8.1 × 10^−7^ C at 150 kV/mm at 60 °C and 80 °C. The Q was 6.8 × 10^−6^ C at 470 kV/mm at 60 °C, as shown in Figure 10b, and 9.6 × 10^−6^ C at 390 kV/mm at 80 °C, as shown in Figure 10c. At 100 °C and 120 °C, Q increased tremendously. The Q was, respectively, 1.3 × 10^−5^ C and 1.9 × 10^−5^ C under 150 kV/mm at 100 °C and 120 °C. The Q was 1.5 × 10^−5^ C at 390 kV/mm at 100 °C, and 6.6 × 10^−5^ C at 270 kV/mm at 120 °C. This indicates that a large amount of space charge can be accumulated in the PP film under a high direct current (DC) field at 40–120 °C, accelerating the failure of its electrical properties. The space charges accumulated during electrical stress were far greater than the space charges that migrated away during short-circuit measurement. A large amount of space charge existed in the films at the end of the short-circuit measurement [34].

**Figure 11:**
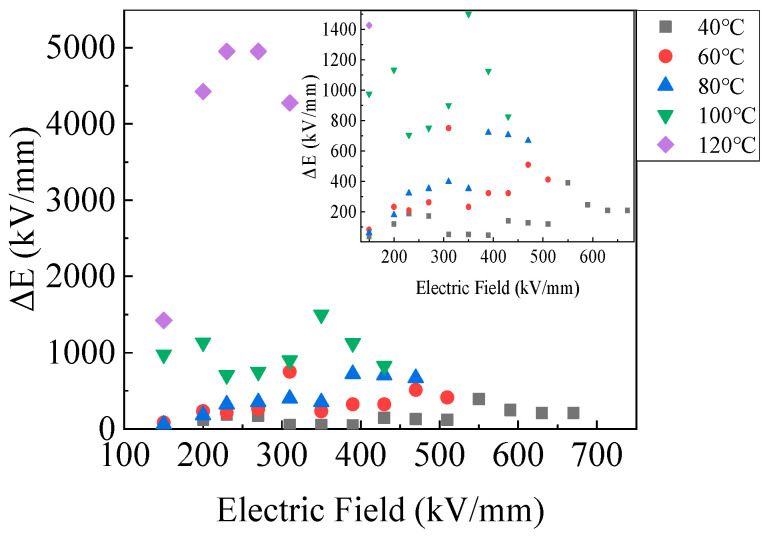
The electric field distortion of the PP film after charging and discharging under different electric fields.

The electric field distortion (ΔE) of the PP film derived from charge accumulation caused by charging and discharging for 7200 s is summarized in Figure 11. The ΔE was calculated based on the Poisson equation, which displayed a very large difference at 40–120 °C. It is obvious that ΔE was relatively small under different electric fields at 40 °C. The ΔE was 39.75 kV/mm T 150 kV/mm for 7200 s. When the electric field was 230 kV/mm for 7200 s, the ΔE increased to 187.5 kV/mm. The applied electric field before breakdown was 630 kV/mm, which contributed to the ΔE to 210 kV/mm. The ΔE was 82.5 kV/mm at 150 kV/mm at 60 °C. As the electric field increased, the ΔE rose. The continuously increasing 470 kV/mm increased the ΔE to 510 kV/mm. At 80 °C, the initial ΔE at 150 kV/mm was 60.8 kV/mm, almost the same as at 60 °C. But the steeper curve confirms the larger ΔE with electric field increasing. The ΔE at 150 kV/mm at 100 °C and 120 °C was, respectively, 975 kV/mm and 1425 kV/mm, which was several times larger than breakdown field strength, and it increased with electric field increasing. It was assumed that local breakdown occurred inside the PP film once initial voltage was applied at 100 °C and 120 °C.

## 5. Conclusions

We comprehensively measured charging and discharging currents versus time. Furthermore, we studied the electric field and temperature dependence of charging and discharging currents and calculated the space charge accumulation. The following conclusions can be drawn:

(1) The charging and discharging currents reached stable values more quickly with higher temperatures and electric fields. The stabilization values of charging currents increased by an order of magnitude from 150 kV/mm to breakdown field at a constant temperature and from 20–80 °C to 100–120 °C under the same electric field. The discharging current stabilization values decreased by an order of magnitude compared with those of charging currents.

(2) The larger the electric field, the more space charges were trapped in the same trap energy level of the PP film at a constant temperature. High temperatures contributed space charge to trap in deeper traps in PP film through the hopping conduction mechanism. 

(3) Carrier mobility did not vary noticeably with the electric field but increased slowly with an elevated temperature. For example, it was almost 10^−18^ m^2^/V·s at 40 °C and 7000 s. It was about 10^−17^ m^2^/V·s at 120 °C at 7000 s.

(4) The charge accumulation that had occurred before the breakdown had increased significantly (by an order of magnitude) with the electric field at the same temperature. At 40–80 °C, the charge accumulation increased with the temperature, for which all the values were the same order of magnitude (10^−6^ C). But it increased to another larger magnitude (10^−5^ C) at 100–120 °C. The electric field distortion derived from charge accumulation was larger at 100–120 °C than at 40–80 °C.

In conclusion, whether polypropylene film or capacitors, operation at relatively low electric fields and temperatures below 80 °C was more favorable to the insulation properties. When the temperature and electric field increased, the charge transport process in PP film was accelerated, and the charge was trapped in deeper traps. Excessively high temperatures or electric fields may cause large space charge accumulation and electric field distortion in the PP film, which can lead to insulation failure, affecting the performance of capacitors, or, in more serious cases, causing the failure of the capacitors. This work provides important support for the rational design of PP film and capacitors operating under extreme electric fields and high temperatures.

## Data Availability

All the research data is in the article.
